# Inflammasome inhibitors: promising therapeutic approaches against cancer

**DOI:** 10.1186/s13045-019-0755-0

**Published:** 2019-06-26

**Authors:** Shengchao Xu, Xizhe Li, Yuanqi Liu, Yu Xia, Ruimin Chang, Chunfang Zhang

**Affiliations:** Department of Thoracic Surgery, Xiangya Hospital of Central South University, Changsha, 410008 Hunan People’s Republic of China

**Keywords:** Inflammasome inhibitors, Therapeutics, Cancer

## Abstract

Inflammation has long been accepted as a key component of carcinogenesis. During inflammation, inflammasomes are potent contributors to the activation of inflammatory cytokines that lead to an inflammatory cascade. Considering the contributing role of inflammasomes in cancer progression, inflammasome inhibitors seem to have a promising future in cancer treatment and prevention. Here, we summarize the structures and signaling pathways of inflammasomes and detail some inflammasome inhibitors used to treat various forms of cancer, which we expect to be used in novel anticancer approaches. However, the practical application of inflammasome inhibitors is limited in regard to specific types of cancer, and the associated clinical trials have not yet been completed. Therefore, additional studies are required to explore more innovative and effective medicines for future clinical treatment of cancer.

## Background

Inflammasomes are multimeric proteins that promote immune responses and the programmed cell death process known as pyroptosis by the activation of caspase-1 in response to pathogen-associated molecular patterns (PAMPs) or danger-associated molecular patterns (DAMPs). The inflammasome was first described by the team of Dr. Jürg Tschopp in 2002 [1], and this group discovered the features of the inflammasome in cold-associated periodic syndromes, gout and type 2 diabetes in follow-up studies [2]. However, emerging evidence indicates that inflammation triggered by viral or microbial infection plays a crucial role in tumorigenesis [3]. Inflammation associated with cancer progression is triggered by innate immune cells, including dendritic cells, natural killer (NK) cells, and macrophages [4]. Immune cells activated by tumors or tumor components might lead to antitumor immune responses through the recruitment of cytotoxic T cells or the promotion of cancer development by creating a proinflammatory context [5]. A key mechanism inducing inflammation in immune cells is orchestrated by the inflammasome. The activation of the inflammasome leads to the production of interleukin 1β (IL-1β) and interleukin 18 (IL-18) and initiates the programmed cell death process known as pyroptosis [6]. In view of the correlation between the inflammasome and cancer development, inflammasome inhibitors have drawn worldwide attention in the development of novel approaches for cancer treatment.

**Fig. 1:**
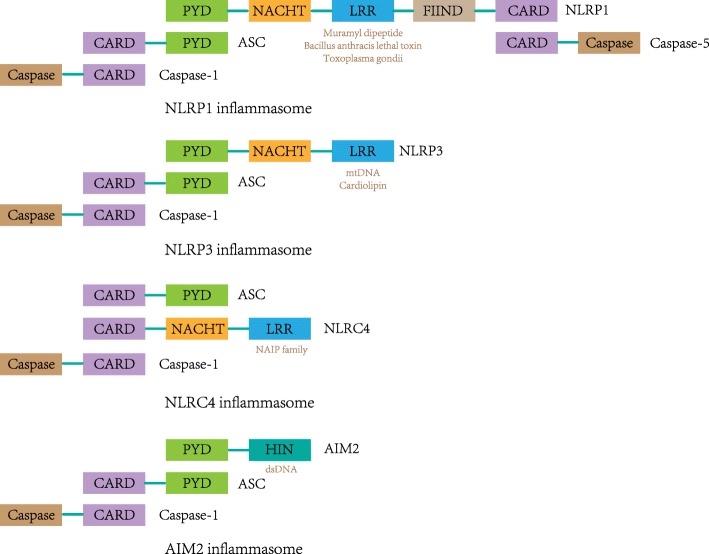
Structures of the NLRP1, NLRP3, NLRC4, and AIM2 inflammasomes. NLRP1 interacts with ASC and caspase-1 via an N-terminal PYD and binds caspase-5 to the complex via the C-terminal CARD. Muramyl dipeptide, *Bacillus anthracis* lethal toxin, and *Toxoplasma gondii* induce the activation of the NLRP1 inflammasome. NLRP3 interacts with ASC through an N-terminal PYD domain, which recruits caspase-1. NLRP3 is activated by the recognition of mtDNA and cardiolipin. The NLRC4 inflammasome is activated by the NAIP family, and it can recruit caspase-1 directly via its CARD in an ASC-independent manner. However, it remains unclear how ASC interacts with the NLRC4 inflammasome complex. The AIM2 inflammasome recruits ASC and caspase-1 through its N-terminal PYD domain and is activated by direct binding with dsDNA via its HIN domain

Inflammasomes consist of NOD (nucleotide oligomerization domain)-like receptors (NLRs), an apoptosis-associated speck-like protein containing a caspase recruitment domain (ASC) and caspase-1. The NLRs generally comprise a leucine-rich repeat (LRR) at the C-terminus, a caspase recruitment domain (CARD) or pyrin domain (PYD) at the N-terminus, and a nucleotide-binding oligomerization domain (NACHT) in the middle. The LRR domain is a sensor that receives signals from PAMPs and DAMPs, while the CARD or PYD interacts with the PYD domain in ASC [1]. Inflammasomes are categorized by their different NLRs such as NLRP1, NLRP3, NLRC4, and AIM2 for identification (Fig. 1). In comparison with NLRP3, NLRP1 has additional function-to-find domain (FIIND) and CARD domains at the N-terminus, which interact with caspase-5 [7]. Inflammasomes lacking a PYD, such as NLRC4, can directly bind with caspase-1 through the C-terminal CARD domain in an ASC-independent manner. However, it remains unclear how ASC interacts with the NLRC4 inflammasome complex [8, 9]. AIM2 consists of a C-terminal HIN domain and an N-terminal PYD, through which AIM2 can recruit ASC and caspase-1 to form the AIM2 inflammasome [10].

As a key regulator in inflammation, inflammasomes can activate inflammatory cytokines such as IL-1β and IL-18 in response to PAMPs or DAMPs [11]. The NLRP1 inflammasome is activated by muramyl dipeptide, *Bacillus anthracis* lethal toxin, and *Toxoplasma gondii*, and the NLRP3 inflammasome can be activated by the combination of mtDNA and cardiolipin. Recognition of NAIP family members induces the activation of the NLRC4 inflammasome, whereas the AIM2 inflammasome can be activated by direct binding with dsDNA via its HIN domain [12]. Inflammasome activation induces the production of IL-1β, which has been implicated in metabolic disorders. Studies have shown that IL-1β plays critical roles in type 2 diabetes and gout and that the blockade of IL-1β exhibits high efficacy in clinical trials [13, 14]. Moreover, the inflammasome is increasingly suspected of playing critical roles in autoinflammatory disorders, Alzheimer’s disease, and cancer [15].

In this review, we summarize the structures and functions of inflammasomes and the signaling pathway that activates inflammasomes, which induce inflammatory cascades. In this regard, multiple drugs that inhibit inflammasomes have been generalized as novel medications against various types of cancer, and some are worthy of further study. Finally, we list some inflammasome inhibitors whose anti-inflammatory activities are well proven. However, their antitumor activities remain to be discovered. Considering the correlation between inflammation and cancer development, these drugs are expected to be innovative therapeutics for cancer treatment.

## Inflammasome signaling pathway

Canonical inflammasome activation requires two signals. The first signal, defined as priming, is the recognition of a DAMP or PAMP by pattern recognition receptors (PRRs), such as Toll-like receptors (TLRs) and NLRs, which initiate innate and adaptive immunity. Here, we focus on the role of NLRs due to their necessity in forming the inflammasome complex. In response to the recognition of a PAMP or DAMP, NLRs oligomerize into homo- or heteroproteins and activate NF-κB. The activation of NF-κB induces the mRNA and protein expression of pro-IL-1β and pro-IL-18 [16]. The second signal is triggered by diverse stimuli activating NLRs, leading to the assembly of inflammasomes via the CARD domain in ASC, which can recruit caspase-1 and interact with NLRPs [17]. When caspase-1 is associated with NLRPs and ASC, the inflammasome complex promotes autocatalytic cleavage of caspase-1, forming the active form of the caspase-1 enzyme [18]. Active caspase-1 can activate pro-IL-1β and pro-IL-18, which are inflammatory cytokines that generate inflammatory responses [19]. Moreover, active caspase-1 also leads to the programmed cell death process termed pyroptosis in certain circumstances. In contrast to apoptosis, pyroptosis results in the rupture of the plasma membrane and release of DAMP molecules such as ATP and IL-1α into the extracellular space, which recruits more immune cells and further promotes the inflammatory cascade [20].

In contrast to the canonical pathway, the noncanonical pathway engages caspase-11 or caspase-8. In response to pathogens such as *Escherichia coli*, *Citrobacter rodentium*, or *Vibrio cholera*, caspase-11 is activated, leading to caspase-1-independent macrophage cell death and caspase-1-dependent IL-1β and IL-18 secretion [21]. In addition, the combination of fungi, Mycobacteria, and the dectin-1 receptor can trigger the formation of the noncanonical inflammasome complex consisting of MALT1, caspase-8, and ASC, which induces the activation of pro-IL-1β and the maturation of IL-1β [22].

Both the priming and the activation of the inflammasome are regulated by a deubiquitination mechanism. The application of G5, an inhibitor of deubiquitination, suggests the involvement of a deubiquitinating enzyme in the activation of the NLRP3 inflammasome. BRCC3, a deubiquitinating enzyme, was identified to regulate NLRP3 ubiquitination by targeting the LRR domain [23]. Moreover, IL-1R-associated kinase (IRAK) plays a critical role in NLRP3 priming by regulating NLRP3 deubiquitination, as demonstrated by using deficient mouse models. IRAK1 and IRAK4 interact with MyD88 in the transcriptional priming phase, whereas IRAK1 regulates posttranslational NLRP3 activation via the TRIF pathway [24]. Notably, the involvement of mitochondrial reactive oxygen species (mtROS) continues to be debated [25]. The deubiquitination pathway mediated by MyD88 is demonstrated to be mtROS dependent and can be inhibited by antioxidants; however, signaling by ATP can also induce the deubiquitination of NLRP in an mtROS-independent manner [26].

There also exist two molecules—heat shock protein 90 (HSP90) and the ubiquitin ligase-associated protein SGT1—that are important for NLRP3 activation. Downregulation of SGT1 expression by siRNA or chemical inhibition of HSP90 can significantly decrease inflammasome activity, leading to repression of NLRP3-mediated gout-like inflammation in mice. Moreover, the interaction of these molecules with NLRP3 is suggested to maintain NLRP3 in an inactive state. Once activating signals are detected, HSP90 and SGT1 dissociate from NLRP3, allowing inflammasome oligomerization [27].

Mitochondrial dysfunction is also involved in inflammasome activation. After the recognition of activating signals such as ATP or LPS, mitochondrial DNA is released into the cytosol and then bound directly by the NLRP3 inflammasome, leading to the activation of the inflammasome and maturation of caspase-1 [28]. During the binding of mtDNA and NLRP3, mitochondrial antiviral signaling protein (MAVS) and mitofusin2 (Mfn2) are thought to be implicated in NLRP3 activation; however, the actual interactions and functions of these proteins are not yet known [29, 30].

Thus, inflammasomes are essential in the immune system, and their roles in the activation of inflammation are incontrovertible. During inflammation, the stimulated inflammasome rapidly produces activated caspase-1, leading to cell pyroptosis and the release of inflammatory cytokines. Inflammatory cytokines are believed to participate in the processes of angiogenesis, metastasis, and epithelial-to-mesenchymal transition activation, which substantially contributes to cancer development [31]. Concerning the relationship between inflammation and cancer, the inflammasome appears to play a detrimental role in cancer due to its proinflammatory activity. However, the direct effect of inflammasome activation on cancer promotion remains controversial.

## Contrasting roles of inflammasomes in cancer

Previous studies have shown that the activated inflammasome plays contrasting roles in cancer promotion and therapy [32]. A protective role for the inflammasome has mainly been observed in colitis-associated cancer. Dextran sulfate sodium (DSS) and azoxymethane (AOM) plus DSS mouse models show increases in the incidences of acute and recurrent colitis-associated cancer in mice lacking inflammasome genes, which are correlated with the levels of IL-1β and IL-18 at the tumor site [33–36]. Moreover, bone marrow reconstitution experiments have demonstrated increased inflammation and tumorigenesis in colitis-associated colon cancer in mice lacking NLRP1 [37]. Additionally, caspase-1-deficient mice have enhanced tumorigenesis as a result of increasing colonic epithelial cell proliferation in the early stage of cancer and reducing apoptosis in advanced colon cancer [38]. In other malignancies, NLRC4 suppresses the tumor growth of melanoma by stimulating tumor-associated macrophages and generating protective T cells [39]. In addition, elevating AIM2 expression by delivering an exogenous AIM2 promotor can significantly inhibit the proliferation and invasion of renal carcinoma [40]. Furthermore, the activation of NLRP1 by serine dipeptidases 8 (DPP8) and DPP9 mediates caspase-1-dependent pyroptosis in human acute myeloid leukemia [41]. This antitumor activity achieved by inhibiting NLRP1 is also exhibited in chronic myeloid leukemia [42].

However, the activation of the inflammasome can also facilitate tumor development. In a mouse model of intravenous injection of B16-F10 melanoma cells, researchers found that mice lacking NLRP3 had a significant decrease in lung metastases compared with wild-type mice and that the pathway was independent of caspase-1 and IL-1β [43]. An analysis of tissue-specific knockout mouse strains fully deficient in ASC used in a chemical-induced skin carcinogenesis model showed that ASC affected tumor proliferation in a dichotomous way: it favored tumor growth via a proinflammatory role in infiltrating cells, while it also limited keratinocyte proliferation and thus helped to suppress tumors [44]. However, ASC protein expression is repressed in metastatic melanoma compared with primary melanoma, and inflammasome-associated caspase-1 and IL-1β are inhibited when the ASC gene is inhibited in primary and metastatic melanoma cells [45]. Moreover, researchers have found that in animal and human breast cancer models, the inflammasome and IL-1β pathway promotes tumor proliferation and migration and that mice lacking inflammasome components exhibit notably suppressed tumor growth and lung metastasis [46]. Additionally, among risk factors for breast cancer, obesity has been associated with a poor clinical prognosis. Studies have found that the activation of the obesity-associated NLRC4 inflammasome drives breast cancer progression [47]. However, in pancreatic ductal adenocarcinoma, studies have demonstrated that the inhibition or deletion of NLRP3, ASC, or caspase-1 decreases tumor growth and metastasis by reprogramming innate and adaptive immunity in the tumor microenvironment [48]. A detrimental role for NLRP3 has also been observed in malignant mesothelioma [49]. AIM2, a subtype of inflammasome, was reported to be a cancer suppressor gene in early years. A recent study showed that AIM2 was highly expressed in non-small cell lung cancer (NSCLC) and promoted tumor development in an inflammasome-dependent manner [50]. As a molecule downstream of the inflammasome, IL-1β has been demonstrated to promote tumor progression by recruiting myeloid-derived suppressor cells, which might inhibit the antitumor immune response [51].

Considering the aforementioned findings, the inflammasome seems to play contrasting roles in cancer development. We hypothesize that different immune responses determine the role of the inflammasome in different types of cancer. In most malignancies, the activation of the inflammasome can lead to either immune surveillance against the tumor or an inflammatory response that promotes cancer development. In colon cancer, the activation of the inflammasome protects the epithelium from cancer invasion. A recent study found that mice deficient in IL-18 and IL-18 receptor but not wild-type mice are highly susceptible to AOM/DSS-induced colon cancer [52]. Considering that DSS induces mucosal damage in the intestinal epithelium, IL-18 secreted during inflammasome activation might be able to maintain the homeostasis of the epithelial barrier, which could account for its antitumor activity. On the other hand, this study showed that epithelial-derived IL-18 could directly interact with CD4 T cells, leading to the suppression of Th17 cell differentiation. However, IL-18 receptor is critical in Foxp3 Treg cells, which mediate the reduction in intestinal inflammation [53]. These findings suggest that the activation of the inflammasome induces the production of IL-18 and that IL-18 then reduces intestinal inflammation by repressing Th17 cells and elevating Treg function. The reduction in inflammation maintains the homeostasis of the intestinal epithelium, leading to the suppression of colon cancer. Further investigations are warranted to verify this hypothesis. The heterogeneity of the inflammasomes in various cancers suggests that inhibitor application should be tailored to the specific situation.

## Antitumor effects of inflammasome inhibitors

**Fig. 2:**
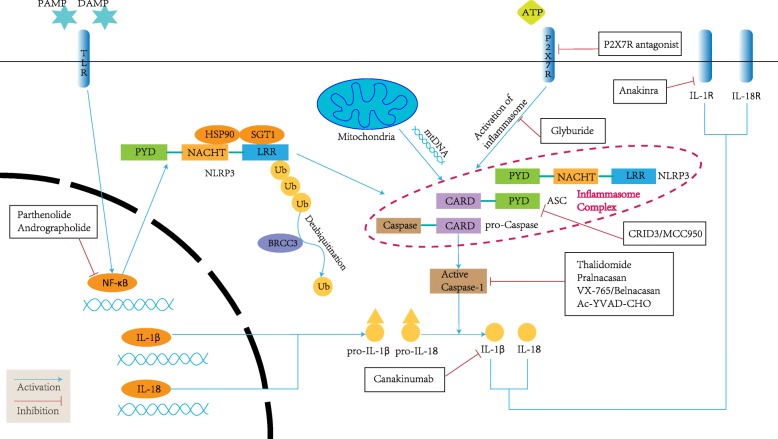
Signaling pathway and inhibitors of inflammasomes. The priming of the inflammasome is initiated by the recognition of a PAMP or DAMP, which mediates the activation of NF-κB. The activation of NF-κB induces the production of NLRP3 and the generation of pro-IL-1β and pro-IL-18. After deubiquitination and combination with mtDNA, NLRP3 interacts with ASC and caspase-1, forming the inflammasome complex. The inflammasome is activated by the recognition of P2X7R, leading to the cleavage of caspase-1. Active caspase-1 then promotes the secretion of IL-1β and IL-18, which is the key to inducing inflammasome-dependent inflammation. Inflammasome inhibitors target the upstream and downstream molecules in the inflammasome signaling pathway. Arrows denote an activating effect, and blunted lines denote targets inhibited by selective compounds

As excessive inflammation induced by the inflammasome can be a detrimental factor in multiple types of cancer, inflammasome inhibitors appear to be a promising approach for cancer prevention and treatment. Currently, many drugs and molecules have been shown to regulate inflammasome activity. However, some of them target the noncanonical signaling pathway of the inflammasome or indirectly affect the functions of the inflammasome by targeting other molecules. Here, we have listed the drugs targeting the canonical signaling pathway of the inflammasome and the antagonists most investigated in cancer treatment (Fig. 2; Table 1).

**Table 1:** Studies and clinical trials of inflammasome inhibitors in cancer

Drug	Target	Effective cancer type	Clinical trials	Reference
Thalidomide	Caspase-1	Prostate cancer	Phase II	[54]
		Multiple myeloma	Phase III	[55]
Anakinra	IL-1R	Melanoma	N/A	[56]
		Breast cancer		[57]
		Multiple myeloma	Phase II	[58]
P2X7R antagonist	P2X7R	Prostate cancer	N/A	[59]
		Pancreatic ductal adenocarcinoma (PDAC)		[60, 61]
		Osteosarcoma		[62]
		Multiple myeloma		[63]
		Head and neck squamous cell carcinoma		[64]
		Colorectal cancer		[65]
		Basal cell carcinoma	Phase I	[66]
Parthenolide	NF-κB	Gastric cancer	N/A	[67]
		Colorectal cancer		[68]
		Pancreatic adenocarcinoma		[69]
		Nasopharyngeal carcinoma		[70]
Andrographolide	NF-κB	Insulinoma	N/A	[71]
		Colorectal cancer		[72–74]
		Breast cancer		[75, 76]
		Multiple myeloma		[77]
Canakinumab	IL-1β	Lung cancer	Phase III (undergoing)	[78]

### Drugs already used in clinical applications

#### Thalidomide

In the past, thalidomide has mainly been used as a sedative or hypnotic drug to treat anxiety, insomnia, gastritis, and tension [79]. The antitumor activity of thalidomide was discovered when it was used for the treatment of erythema nodosum leprosum because of its antiangiogenic properties [80]. However, due to its potential to cause congenital defects, thalidomide analogs have mostly been applied to many types of cancer, including prostate cancer and multiple myeloma.

For the treatment of multiple myeloma, thalidomide has been approved for first-line therapy in combination with other chemotherapeutic drugs [81]. In patients with relapsed myeloma, few therapies are available. However, researchers have found that thalidomide has a practical antitumor effect on patients with advanced myeloma. According to statistics, 10% of patients experience complete or almost complete remission and 32% exhibit a decrease in serum or urine paraprotein levels. In most patients, the percentage of plasma cells in the bone marrow is reduced, and the hemoglobin level is elevated, indicating substantial antitumor activity against myeloma [55]. In a randomized phase II trial, the combination of thalidomide and docetaxel resulted in a significant reduction in the prostate-specific antigen level and an elevation of the median survival rate in patients with metastatic androgen-independent prostate cancer [54]. The mechanism of malignancy control with thalidomide might involve its antiangiogenic activity. Thalidomide was demonstrated to reduce the high levels of certain angiogenic factors, such as fibroblast growth factor (FGF) and vascular endothelial growth factor (VEGF) [82]. Moreover, thalidomide enhances cell-mediated immunity by directly interacting with cytotoxic T cells, which are lethal to tumor cells [83].

However, the application of thalidomide in other carcinomas has not shown significant efficacy. In unresectable hepatocellular carcinoma, thalidomide is tolerated in most patients with gradual dose escalation, but its monotherapeutic activity is modest compared with that of the combined therapy [84]. In a randomized, double-blinded, placebo-controlled trial, 722 patients with advanced non-small cell lung cancer were treated with thalidomide in combination with gemcitabine and carboplatin. The results showed that this treatment regimen did not improve the survival rate but did increase the risk of thrombotic events [85]. Moreover, this outcome was also demonstrated in a phase III clinical trial performed in France, and neuropathy was the most common adverse event observed [86]. Additionally, in patients with metastatic melanoma, the combination of thalidomide and dacarbazine or temozolomide shows limited efficacy. Constipation, peripheral neuropathy, fatigue, edema, and rash are attributed to thalidomide [87, 88].

Overall, thalidomide is widely used in the treatment of multiple myeloma and prostate cancer. Especially in multiple myeloma, the combination of melphalan-prednisone-thalidomide is deemed a standard therapy for patients ineligible for stem cell transplantation [89]. However, its antitumor activity has a moderate effect on other types of cancer.

#### Anakinra

Anakinra is a recombinant form of interleukin-1 receptor antagonist (IL-1Ra), which is commonly applied in the treatment of rheumatoid arthritis and autoinflammatory disease [90].

Previous studies with myeloma cells have shown that anakinra can significantly reduce IL-6 levels but does not increase myeloma cell death. However, a combination therapy of anakinra and dexamethasone induces cell death in myeloma cells [91]. In a study of breast cancer mouse models, anakinra decreased the growth of tumors in the bone and reduced the number of mice with bone metastasis from 90% (placebo) to 40% (treatment) or 10% (preventative). This study indicated that anakinra fails to increase tumor cell death but represses cell proliferation and angiogenesis [57]. Melanoma, the most dangerous type of skin cancer, has a poor prognosis. A study found that anakinra increases M1 macrophage polarization and decreases myeloid-derived suppressor cell numbers in mice with melanoma [56]. In phase II clinical trials, researchers investigated the roles of anakinra and low-dose dexamethasone in patients with smoldering or indolent multiple myeloma. The results showed that anakinra targets the progressed myeloma fraction in vivo and decreases the proliferation of myeloma cells [58]. Then, the antitumor activity of anakinra was mainly mediated by reducing angiogenesis. The administration of anakinra alleviated the levels of CD34-positive blood vessels and significantly reduced the expression of the endothelin 1 gene [57]. Moreover, previous studies have shown that IL-1β elevates the expression of VEGF and VEGF represses the activities of IL-1β [92, 93]. The inhibition of IL-1β by anakinra could obviously suppress the activity of VEGF, leading to an antiangiogenic effect.

Anakinra is usually used as a second-line treatment in rheumatoid arthritis, and subcutaneous injection of anakinra has been approved by the US FDA [94]. However, the antitumor applications of anakinra await further studies.

### Drugs studied in clinical trials

#### P2X7R antagonist

Previous studies have demonstrated that P2X7 is highly expressed in prostate cancer, pancreatic ductal adenocarcinoma (PDAC), head and neck squamous cell carcinoma, colorectal cancer, and papilloma. When the expression of PX27 is downregulated by siRNA, the metastasis and invasion of prostate cancer cells are notably reduced through the PI3K/AKT and ERK1/2 pathways [59]. In PDAC, P2X7R allosteric inhibitor-treated cells exhibited attenuated tumor proliferation and invasion compared to untreated control cells [60]. In addition, extracellular ATP and BzATP, which have relatively high affinities for P2X7R, further impact cell survival and the complex function of P2X7R [61]. Moreover, P2X7R plays an important role in bone tumor growth and functions [95]. In osteosarcoma, P2X7R was proven to facilitate the growth and matrix invasion of tumor cells, which indicates the potential of P2X7R as a therapeutic target [62]. In another bone cancer, multiple myeloma, the activation of P2X7R was also deemed to affect cell necrosis in the RPMI-8226 cell line [63]. Furthermore, the inhibition of P2X7R can lead to decreased invasiveness in A253 cells, which are derived from an epidermoid carcinoma [64]. Since chronic inflammation is a key factor leading to colorectal cancer, P2X7R has been documented as a regulator in inflammatory responses. In colorectal cancer patients, high expression of P2X7R is significantly associated with tumor size and lymph node metastasis [65]. High expression of P2X7 enhances cancer proliferation, migration, invasion, and angiogenesis. Cancer cells can downregulate the expression of P2X7 to avoid apoptosis and use ATP as an invasion-promoting signal [96]. The activation of P2X7 promotes cancer invasion by releasing cathepsin and MMP-9 [97, 98]. Moreover, the P2X7-dependent release of VEGF promotes angiogenesis and contributes to cancer development [99]. These findings suggest that P2X7R antagonists alter the context of tumor cells, leading to the suppression of cancer progression.

In clinical trials, the safety and tolerability of a P2X7 antagonist were assessed in an open-label, phase I study, in which approximately 65% of patients with basal cell carcinoma showed a decrease in lesion area and the most common adverse event was an allergic reaction occurring at the treatment site [66]. These properties warrant additional studies to evaluate the potential of P2X7 antagonists in the treatment of not only skin cancer but also other malignancies.

#### Parthenolide

Parthenolide is a sesquiterpene lactone compound found in the herb named feverfew, which is used as an anti-inflammatory medicine [100]. While NF-κB has been reported to be a key factor regulating a number of genes that are crucial for tumor invasion and metastasis, parthenolide is considered a potential antitumor therapeutic drug that functions by inhibiting the NF-κB signaling pathway [101]. In gastric cancer, parthenolide significantly inhibits tumor cell growth and downregulates the phosphorylation of NF-κB. During a study of combined therapy with parthenolide and paclitaxel, the survival time of patients with gastric cancer was notably prolonged [67]. In addition, in pancreatic adenocarcinoma, tumor cell growth can be inhibited by parthenolide in a dose-dependent manner. After a higher concentration of parthenolide treatment is administered, internucleosomal DNA fragmentation indicative of apoptosis can be observed [69]. In a study of colorectal cancer models, intraperitoneal injection of parthenolide notably inhibited tumor proliferation and angiogenesis. By focusing on the Bcl-2 family in cancer cells, a parthenolide-mediated cell death signaling pathway was investigated and confirmed to be associated with colorectal cancer cell death [68]. In nasopharyngeal carcinoma, parthenolide induces tumor cell death through the NF-κB/COX-2 pathway. Using COX-2 inhibitors or knocking down COX-2 expression with siRNA or shRNA suppresses cancer stem-like cell phenotypes. Parthenolide exerts an inhibitory effect on NF-κB by suppressing both the phosphorylation of IκB kinase and the degradation of IκBα [70]. The mechanisms involved in the antitumor activity of parthenolide, including the inhibition of NF-κB, activation of JNK, activation of p53, and suppression of STAT3, have attracted great interest. Parthenolide sensitizes cancer cells to TNF-α-induced apoptosis by inhibiting NF-κB and activating JNK [102]. The administration of parthenolide can activate p53, leading to a reduction in cancer cell proliferation [103]. Moreover, parthenolide can inhibit the activation of STAT proteins by blocking their tyrosine phosphorylation, which is indispensable for STAT translocation into the nucleus and target gene activation [104].

In practical use, the low solubility and bioavailability of parthenolide limit its potential [105]. However, creating synthetic analogs of parthenolide may be a novel way to address this problem [106]. Currently, a clinical trial of parthenolide is being performed in allergic contact dermatitis [107]. Therefore, additional studies are required to exploit parthenolide as a novel antitumor drug.

#### Canakinumab

Canakinumab is a human monoclonal antibody targeting IL-1β but not IL-1α. In 2009, canakinumab was authorized by the US Food and Drug Administration and European Medicines Agency as a treatment for cryopyrin-associated periodic syndromes [108]. A randomized, double-blinded trial found that compared with controls, canakinumab at a dosage of 150 mg every 3 months led to a notable reduction in the rate of recurrent cardiovascular events [109]. When cancer is considered, canakinumab still deserves recognition. During a randomized, double-blinded, placebo-controlled trial of patients with lung cancers and atherosclerosis, researchers found that canakinumab could significantly decrease lung cancer mortality by targeting the IL-1β innate immunity pathway. It is worth mentioning that this antitumor effect is mostly detected in patients with lung adenocarcinoma or poorly differentiated large cell cancer, whereas meaningful assessments of the effects on patients with small cell lung cancers or squamous cell carcinomas have rarely been performed [78]. The chronic use of aspirin can reduce mortality in colorectal cancer and lung adenocarcinomas due to its anti-inflammatory activity [110, 111], and canakinumab is theorized to combat cancer in a similar way, according to its function of inhibiting the inflammasome [78].

Currently, the application of canakinumab as an antitumor drug is mainly focused on lung cancer. Canakinumab is being studied in phase III clinical trials in non-small cell lung cancer to evaluate its tolerability and efficacy compared with those of a placebo. Consequently, the completion of the clinical trials is warranted to determine whether canakinumab can be used safely and effectively in cancer treatment.

#### Andrographolide

Andrographolide is a labdane diterpenoid that has been isolated from the stem and leaves of *Andrographis paniculata* [112]. Numerous studies have validated the facts that andrographolide can inhibit cell invasion and induce cell death in various types of cancer cells. A recent study showed that andrographolide significantly reduces tumor cell proliferation at both the early stage and advanced stage of insulinoma by targeting the TLR4/NF-κB signaling pathway [71]. In addition, in colon cancer, andrographolide represses cell proliferation, elevates cell apoptosis, and activates caspase-3/9 in SW620 human colon cancer cells by inhibiting NF-κB, TLR4, MyD88, and MMP-9 signaling activation [72]. Among chemotherapeutic drugs, 5-fluorouracil (5-Fu) is the one most commonly used in colorectal cancer. Andrographolide can promote the 5-Fu-induced antitumor effect by repressing the level of phosphorylated cellular-mesenchymal to epithelial transition factor [73]. In colitis-associated cancer, andrographolide inhibits the NLRP3 inflammasome, protecting mice against dextran sulfate sodium-induced colon carcinogenesis [74]. In breast cancer, andrographolide suppresses breast cancer-induced osteolysis by inhibiting the NF-κB and ERK signaling pathway at a relatively low dose and by promoting apoptosis at a relatively high dose. Its antitumor activity correlates with the downregulation of the NF-κB signaling pathway [75, 76]. Moreover, andrographolide reduces proliferation and increases cell apoptosis by downregulating the protein expression of TLR4 and NF-κB in multiple myeloma [77]. The antitumor mechanisms of andrographolide include the inhibition of the NF-κB pathway [113], suppression of cyclins and cyclin-dependent kinases [114], and activation of the p53 protein [114], leading to reductions in cancer cell proliferation, invasion, and angiogenesis.

Clinical trials of andrographolide have mainly focused on inflammatory diseases such as acute upper respiratory tract infections [115, 116], and its antitumor activity has been demonstrated only in vitro. Therefore, more studies are required to investigate its application in cancer treatment.

Overall, clinical application was limited to only anakinra and thalidomide, and other drugs are still under assessment in clinical trials. All of these drugs inhibit the production and activation of inflammasome-associated molecules such as P2X7R, IL-1, NF-κB, and caspase-1, leading to the suppression of the inflammasome. As mentioned above, the antitumor mechanisms of these drugs involve the regulation of the expression of p53, NF-κB, STAT, and VEGF, leading to the suppression of tumor cell proliferation, metastasis, invasion, and angiogenesis. However, the direct interactions of inflammasome inhibitors involved in repressing cancer development are not yet known. Further studies are needed to explore the mechanisms in a more explicit way.

## Potential antitumor drugs

Considering the correlation between inflammation and tumorigenesis, it is rational to expect that antagonists that inhibit the initiation of inflammation could be explored as potential antitumor drugs. In the inflammasome signaling pathway, there are many steps that could be targeted, such as the assembly and activation of inflammasomes, the synthesis of IL-1, and the generation of caspase-1. Several inhibitors targeting the above processes hold promise in developing novel drugs against cancer and are described below.

### Glyburide

Glyburide is an antidiabetic drug in a class of medications known as sulfonylureas, which are commonly used in the therapy of type 2 diabetes [117]. Glyburide was demonstrated to block ATP-sensitive potassium channels in pancreatic B cells [118]. In placental inflammation-associated diseases, trophoblasts can secrete IL-1β through the NLRP3 pathway, which plays an important role in inflammation-associated pregnancy complications, and glyburide offers considerable therapeutic promise as an inhibitor of the NLRP3 inflammasome [119]. Moreover, glyburide was beneficial in human melioidosis in a study of 1160 patients with gram-negative sepsis due to its inhibitory effect on the inflammasome and subsequent suppression of the inflammatory response. Considering the role of the NLRP3 inflammasome in endotoxemia, the data suggest that glyburide can delay lipopolysaccharide (LPS)-induced lethality in mice [120].

However, since glyburide specifically inhibits the NLRP3 inflammasome in vitro, treatment requires the administration of a high dose in vivo, which causes hypoglycemia and is beyond its pharmacological action in type 2 diabetes. A recent finding suggests that the small molecule 16673-34-0, which is an intermediate substrate in the synthesis of glyburide, disrupts the synthesis of the NLRP3 inflammasome and limits infarct size in mouse models of myocardial infarction without affecting glucose metabolism [121]. Therefore, this substrate, which exhibits pharmacodynamics similar to those of glyburide, may be a novel inhibitor of the inflammasome with fewer side effects than glyburide.

### CRID3/MCC950

The cytokine release inhibitory drugs (CRID3), also known as MCC950, are diarylsulfonylurea-containing compounds that inhibit the activation of the NLRP3 inflammasome both in mice in vivo and in human cells in vitro [122]. Researchers have found that CRID3 inhibits the secretion of IL-1β and caspase-1 in response to the NLRP3 and AIM2 inflammasomes but not in response to the NLRC4 inflammasome. In contrast to the NLRP3 inhibitors glyburide and parthenolide, CRID3 can prevent AIM2-dependent pyroptosis. Moreover, the potential target of CRID3 was identified as glutathione S-transferase omega 1 (GSTO1), a protein that has been demonstrated to interact with ASC [123, 124]. In a study of a spontaneous chronic colitis mouse model, MCC950 resulted in significant suppression of IL-1β secretion and activation of caspase-1, indicating a potential novel avenue for treatment of human colonic inflammation diseases [125].

### Pralnacasan

Pralnacasan is an orally absorbed nonpeptide compound that inhibits interleukin 1β converting enzyme (ICE), which is also known as caspase-1 [126]. ICE exists in the plasma membrane of monocytic cells where it activates the precursors of IL-1β and IL-18 into their active forms. This process is considered to be downstream in the inflammasome signaling pathway [127, 128]. In a collagenase-induced osteoarthritis mouse model, pralnacasan has been demonstrated to reduce joint damage, indicating its potential as a disease-modifying drug for the therapy of osteoarthritis [129]. In dextran sulfate sodium (DSS)-induced murine colitis models, pralnacasan is able to ameliorate dextran sulfate sodium-induced colitis with almost no side effects. This process is probably mediated by the repression of the inflammatory cytokines IL-1β and IL-18 [130]. Researchers found that IL-18 mRNA and TNF-α mRNA levels were elevated in DSS-induced colitis, and the administration of pralnacasan significantly reduced the expression of IL-18 mRNA but did not affect the expression of TNF-α mRNA. Therefore, the therapeutic approach of combining TNF-α expression-reducing substances with pralnacasan appears to be a promising idea [131].

### VX-765

VX-765, also known as belnacasan, is an inhibitor that decreases the activity of caspase-1. A study showed that the administration of VX-765 in rat models significantly reduced the number of seizures and delayed the time to seizure onset [132]. The same anticonvulsant effect of VX-765 has been exhibited in mice models in a dose-dependent manner [133]. Moreover, the application of VX-765 stops the accumulating deposition of amyloid β, indicating its potent therapeutic activity in Alzheimer’s disease [134]. In addition to its inhibitory effect on nervous system disease, VX-765 has also been proven to reduce infarct size in a rat model of myocardial infarction. In combination with an antiplatelet P2Y12 inhibitor, VX-765 exhibited a highly protective function when myocardial infarction occurred [135, 136].

Currently, clinical trials of VX-765 are mainly studying the treatment of epilepsy. A randomized, double-blinded, placebo-controlled phase II study of VX-765 in patients with treatment-resistant partial epilepsy was completed, and the results showed no statistically significant difference between the VX-765 group and the placebo group [137]. Consequently, a longer duration study is warranted to measure the clinical efficacy of VX-765.

### Ac-YVAD-CHO

Ac-YVAD-CHO is a polypeptide with a sequence homologous to the known sequences of caspase substrates, which accounts for its ability to inhibit the activation of caspase-1 [138, 139]. Researchers have used Ac-YVAD-CHO as a therapeutic intervention in pancreatic carcinoma cells, and they found that the inhibition of caspase-1 leads to cell apoptosis. Moreover, according to their observations, caspase-1 was directly involved in antiapoptotic processes in pancreatic cancer [140]. Additionally, the administration of Ac-YVAD-CHO has been demonstrated to induce remission in rats with endotoxemia by decreasing the secretion of IL-1β and IL-18 [141].

Overall, caspase-1 and IL-1β, molecules downstream of the inflammasome, play major roles in the generation of inflammation, and the drugs mentioned above are commonly used in the treatment of inflammation-associated disease because they can reduce the functions of caspase-1 and IL-1β. However, their applications in cancer therapy remain unknown. Thus, further investigations are warranted to characterize the antitumor activities of these potent inflammasome inhibitors.

## Conclusions

The role of the inflammasome in cancer development has received increasing attention in recent years. During the progression of cancer, excessive inflammation stimulated by the inflammasome is the generally accepted hypothesis explaining the detrimental effect of inflammasomes on multiple forms of cancer. In the downstream course of the inflammasome pathway, IL-1β and IL-18 are activated by caspase-1 to generate an inflammatory response. Therefore, drugs that can downregulate the functions of these cytokines seem to have therapeutic activities in inflammation-associated diseases.

In various in vitro experiments, inflammasome inhibitors have been shown to attenuate the proliferation and invasion of cancer cells. However, their antitumor activities are limited to specific types of cancer. In terms of practical applications, the clinical trials studying inflammasome inhibitors have mainly focused on multiple myeloma, in which thalidomide and anakinra are well studied. Otherwise, inflammasome inhibitors are mostly utilized in inflammatory diseases such as osteoarthritis, rheumatoid arthritis, and colon colitis. Considering the limited application of inflammasome inhibitors in cancer treatment, we are looking forward to more broad-spectrum and effective antitumor drugs. Several of the inflammasome inhibitors detailed above have been demonstrated to have the function of reducing inflammatory responses, indicating that inflammasome inhibitors could be novel candidates for the treatment of malignancies in which inflammation is involved as a major contributor.

The correlation between inflammasomes and cancer provides a promising approach for cancer therapy. The contrasting roles of inflammasomes in different cancers suggest the need for specific strategies when inhibitors are applied in cancer treatment. However, the inappropriate administration of inflammasome inhibitors might result in the repression of antitumor immunity and enhanced infection susceptibility and deterioration in autoinflammatory diseases. Consequently, the application of inflammasome inhibitors must be tailored to the specific type of cancer, and further studies are warranted to characterize the antitumor effects of these drugs.

## Data Availability

Not applicable

## Abbreviations

AIM2: Absent in melanoma 2
AOM: Azoxymethane
ASC: Apoptosis-associated speck-like protein containing a caspase recruitment domain
CARD: Caspase recruitment domain
CRID3: Cytokine release inhibitory drugs
DAMP: Danger-associated molecular pattern
dsDNA: Double-stranded DNA
DSS: Dextran sulfate sodium
FIIND: Function-to-find domain
GSTO1: Glutathione S-transferase omega 1
HIN: Hematopoietic interferon-inducible nuclear
ICE: Interleukin 1β converting enzyme
IL-18: Interleukin 18
IL-1β: Interleukin 1β
JNK: c-Jun N-terminal kinase
LPS: Lipopolysaccharide
LRR: Leucine-rich repeat
mtDNA: Mitochondrial DNA
MyD88: Myeloid differentiation factor 88
NACHT: N-terminus and a nucleotide-binding oligomerization domain
NAIP: NLR family, apoptosis inhibitory protein
NLRs: Nucleotide oligomerization domain-like receptors
NOD: Nucleotide oligomerization domain
PAMP: Pathogen-associated molecular pattern
PDAC: Pancreatic ductal adenocarcinoma
PYD: Pyrin domain
STAT: Signal transducers and activators of transcription
